# Bridging the Gap between Affective Well-Being and Organizational Citizenship Behavior: The Role of Work Engagement and Collectivist Orientation

**DOI:** 10.3390/ijerph16224503

**Published:** 2019-11-15

**Authors:** Jia Xu, Baoguo Xie, Beth Chung

**Affiliations:** 1 School of Political Science and Public Administration, Wuhan University, Wuhan 430072, China; jiaxu@whu.edu.cn; 2 School of Management, Wuhan University of Technology, Wuhan 430070, China; 3 Fowler School of Business, San Diego State University, San Diego, CA 92182, USA; bchung@sdsu.edu

**Keywords:** affective well-being, work engagement, collectivist orientation, organizational citizenship behavior, conservation of resources theory

## Abstract

Workplace well-being has received considerable attention over the past decade. Relative to the positive relationship between affective well-being and in-role performance, the relationship between affective well-being and extra-role performance has received little empirical attention. The purpose of this study was to examine the relationships among affective well-being, work engagement, collectivist orientation, and organizational citizenship behavior. Specifically, we tested this model with a sample of 264 employees from a telecom company in China. We found that: (1) affective well-being was the positive predictor of organizational citizenship behavior (*B* = 0.482, *p* < 0.001); (2) work engagement mediated the relationship between employee affective well-being and organizational citizenship behavior (indirect effect = 0.330, *p* < 0.001); and (3) collectivist orientation moderated the relationship between affective well-being and work engagement (*B* = 0.113, *p* < 0.01) and affective well-being and organizational citizenship behavior (*B* = 0.084, *p* < 0.05). Our discussion highlights the benefits of understanding the role of work engagement and cultural values with regard to the relationship between affective well-being and organizational citizenship behavior.

## 1. Introduction

Employee well-being has been one of the hottest topics for organizational behavior researchers and practicing managers since the rise of positive occupational health psychology (POHP) [1]. Interest is strong because there is consensus among experts in the field that the promotion and cultivation of healthy workers and workplaces is of critical importance for maximizing organizational performance [2]. Employee affective well-being not only enhances employee health and quality of life [3,4], but also results in greater business-unit customer loyalty, higher productivity, and lower rates of turnover [5,6].

However, some limitations in previous research on employee affective well-being should be noted. First, although associations between affective well-being and in-role performance are well established [7], organizational scholars have so far largely neglected potential effects of affective well-being on extra-role performance, such as organizational citizenship behavior. Second, although affective well-being has been shown to have a significant predictive effect on employee outcomes, the mechanisms through which affective well-being influences outcomes such as organizational citizenship behavior has received less attention. Last, it is unclear whether workplace well-being is equally important for individuals with different cultural values. As such, identifying the factors that account for individual differences in the well-being to performance relationship is an important issue both for organizational research and for human resource practice. Thus, through a two-wave data collection effort, the current research addresses these issues.

This research makes several contributions to the literature. First, this paper contributes to the well-being literature by investigating the relationship between employee affective well-being and organizational citizenship behavior. As economic globalization continues to grow, traditional employee duties and responsibilities can change frequently; traditional role performance is no longer sufficient to stay competitive [8]. Instead, organizational citizenship behavior, as a voluntary behavior, is also needed and contributes to organizational performance, organizational productivity, and organizational efficacy [9,10]. Investigating the relationship between well-being and organizational citizenship behavior is critical for managers who seek to enhance employees’ organizational citizenship behavior. Second, we extend the well-being literature by using the Hobfoll’s Conservation of Resources theory [11] to better understand how affective well-being relates to organizational citizenship behavior. Conservation of Resources theory is highly apposite for understanding the relationship between affective well-being and organizational citizenship behavior because affective well-being is likely to help employees conserve and develop resources to enhance work engagement which, in turn, is related to organizational citizenship behavior. Third, several studies have shown that cultural values influence employee resource allocations [12,13]. Similarly, we are interested in whether having a collectivist orientation, a typical Chinese cultural value, moderates the relationship between affective well-being and work engagement and affective well-being and organizational citizenship behavior. A collectivist orientation has long been found to influence how employees think and feel about their organization [14]; knowledge about the moderating role of a collectivist orientation can provide insight into the values that interact to affect outcomes.

**Figure 1:**
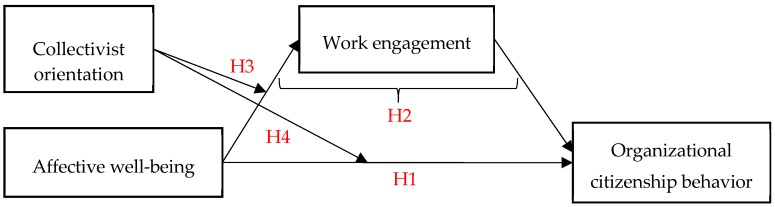
Theoretical model.

In sum, we expect employee affective well-being to relate positively to organizational citizenship behavior directly and via work engagement; we also examine the role of having a collectivist orientation as a moderator. Figure 1 summarizes our theoretical framework.

## 2. Literature Review and Hypothesis Development

### 2.1. Affective Well-being, Work Engagement, Collectivist Orientation, and Organizational Citizenship Behavior

Well-being is an umbrella term for different concepts related to wellness. Well-being has both physical and psychological definitions [15]. Affective well-being or psychological well-being as a prevalent notion in the well-being literature is defined as a state where a person is happy with their life and work. It has often been depicted as representing an individual’s affective state including experiences of more positive emotions and less negative emotions [16,17]. Employee affective or psychological well-being is important to organizations because it has been shown to be positively related to creativity [18] and job performance [7] and negatively related to workplace turnover [6]. In a recent comprehensive book on well-being, David et. al. [15] concluded that a happy worker is a productive worker, but little is known about the relationship between affective well-being and organizational citizenship behavior, an important form of extra-role performance. How and why affective well-being impacts employee organizational citizenship behavior deserves further exploration.

Work engagement is identified as a motivational construct that is characterized by vigor, dedication, and absorption [19]. Vigor is defined as having a high energy level, increased mental resilience, and the willingness to invest effort in one’s work. Dedication is experienced and the individual has a sense of pride, significance, challenge, and enthusiasm at work. Absorption is characterized as being deeply immersed in one’s work whereby time passes quickly, and one finds it difficult to detach oneself from work. Previous research has found that work engagement enables employees to invest their cognitive, physical, and emotional resources to go the extra mile and help the organization to be more productive and efficient [20,21]. Engaged employees immerse themselves more fully into their work and feel a lot more connected to their work.

Collectivist orientation is a well-established cultural construct that has been linked broadly to people’s emotions, motivation, and behavior [22]. At a psychological level, a collectivist orientation is defined as the degree to which individuals hold a general orientation toward group goals, group norms, the well-being of the group and its members, and a tendency toward cooperation in the workgroup [23,24,25]. Scholars have demonstrated that one’s cultural orientation may affect the use or effectiveness of personal resources in the work context; employees with collectivist values are more willing to suppress personal goals for the good of the whole and for contributions made to the effective functioning of the organization [26]. Accordingly, we believe that a collectivist orientation may influence relationships between personal resources and work outcomes.

Organizational citizenship behavior (OCB) is defined by Organ [8] as “individual behavior that is discretionary, not directly or explicitly recognized by the formal reward system, and that in the aggregate promotes the effective functioning of the organization” p. [4]. Organizational citizenship behavior, also known as the “good soldier syndrome”, includes punctuality, helping co-workers, volunteering, as well as the tendency to resist undesirable behaviors such as expressing resentment, cynicism, and carp at others. Organizational citizenship behavior has been explored and researched by scholars for more than two decades and it continues to be a high priority for scholars because it has a positive impact on organizational success through improvement in work effectiveness, job satisfaction, organizational commitment, and leader behavior. A review of the organizational citizenship behavior literature suggests that there are clear relationships between dispositional, attitudinal, motivational, and contextual factors and organizational citizenship behavior [27]. Although this body of work is extensive, the preponderance of research has examined dispositional and attitudinal predictors of organizational citizenship behavior while emotional factors (such as affective well-being) have been largely neglected. 

### 2.2. Conservation of Resources (COR) Theory

As a theory of motivation, the key proposition of Conservation of Resources (COR) theory is that people are motivated to conserve their current resources and acquire new resources because a loss of resources may bring work stress and burnout. Resources are loosely defined as anything people value, such as objects, states, conditions, and energies [28]. From a COR perspective, employees are confronted with loss or a threat of loss of personal resources when job demands are placed on them. The more resources employees have, the more likely they will use productive work behaviors to gain more resources. Conversely, the less resources employees have, maladaptive coping behaviors will be used to protect current resources.

In the Conservation of Resources theory, affective well-being can act as a key resource. There is evidence that various positive emotional experiences are related to job performance. For instance, Parker et al. found that positive emotional experiences were positively related to creativity [29]. Recent research has proposed the possible role of psychological well-being as a resource capable of assisting employees to better deal with various job demands, while also helping to protect them from further resource depletion [30,31]. Thus, the Conservation of Resources theory offers a theoretical explanation by linking affective well-being with the development of resources for exhibiting long-term positive behaviors.

### 2.3. Hypotheses Development

The personal resource building functions described by the Conservation of Resources theory are particularly valuable for understanding how affective well-being affects organizational citizenship behavior. On the one hand, affective well-being is likely to help one to conserve and develop personal and job resources. Unlike negative emotions, which deplete employees’ resources, affective well-being motivates employees to seek social support and learn new things at work [32]. Affective well-being has also been shown to be associated with developing adaptive personal resources in a number of domains, including psychological and physical health [4,33]. Chen and colleagues [34] found affective well-being’s vital contribution to resilience, an important psychological resource. Moreover, affective well-being was also found to be related to job resources. For example, Ilies and Judge [35] found that employees with affective well-being tend to set higher goals, which motivated them to create more job resources to achieve those goals. Thus, we propose that affective well-being will help one to conserve and increase personal and job resources.

One the other hand, organizational citizenship behavior necessitates employees to invest personal and job resources in behaviors that are not required by the job [10]. When employees’ activities extend beyond the prescribed job duties, they may find that they need more time and energy to fulfill their work obligations. For example, helping colleagues with work overload can add to one’s personal workload thereby creating individual costs from taking on multiple responsibilities. Recent research has also shown that engaging in organizational citizenship behaviors can be draining and depleting; good citizens can become drained as a result of their engagement in such behavior [36]. Organizational citizenship behavior is time consuming and can impede employees’ task progress [37,38]. Thus, affective well-being can be conceived of as adding value in aiding the acquisition of desirable personal and job resources in order to enhance organizational citizenship behavior.

**Hypothesis 1** **(H1).** *Affective well-being is positively related to organizational citizenship behavior.*

Although consistent positive links between affective well-being and work outcomes have been shown, research exploring the underlying process remains unclear. We argue that affective well-being should lead to a higher level of organizational citizenship behavior through enhanced work engagement. Work engagement refers to an individual’s work state where one is fully invested at work [18]. As this definition suggests, cognitive, psychological, and physical resources often need to be invested in work. Previous research has clearly highlighted the importance of affective well-being at work; more recent approaches have focused on affective well-being as an emotional resource to help one to engage at work [39,40]. Affective well-being not only directly leads to work engagement, but also indirectly by increasing cognitive, psychological, and physical resources, which are important predictors of work engagement [41]. Findings have shown that affective well-being motivates people to accomplish their work and helps to increase personal and social resources [42]. For example, teachers’ affective well-being has a longitudinal effect on their work engagement even at a later date, such as after 6 months [43]. A diary study also found that employees’ affective well-being leads to a state of vigor, dedication, and absorption [44].

Work engagement has been shown to be a consistent, robust predictor of organizational citizenship behavior. People who are highly engaged in their jobs are in a motivational state of fulfillment; they are not easily fatigued and are willing to allocate personal resources to role performance and provide discretionary effort [44]. According to the Conservation of Resources theory, engaged employees not only use personal resources to meet the task and social demands of work roles, but they are also more likely to evolve and expand their roles by investing more resources which contribute to the growth of the organization. Halbesleben et.al. [45] found that work engagement is significantly positively related to organizational citizenship behavior. Ariani [20] also found that employees who are highly engaged in their work tend to engage in constructive and responsible behavior at work. Therefore, on the basis of previous theoretical and empirical research, we developed the following hypothesis:

**Hypothesis 2** **(H2).** *Work engagement mediates the relationship between affective well-being and organizational citizenship behavior.*

Within the Conservation of Resources theory, there are individual differences in terms of how people react to the resource-based processes of loss, threat, and investment [46]. We proposed that a collectivist orientation is an important moderator of the relationship between affective well-being and work engagement. An individual’s collectivist orientation reflects how hard s/he will work for team goals, which also influences the extent to which these individuals have to draw upon their limited resources. According to the Conservation of Resources theory, there is a limit to the amount of energy and resources available to individuals, thus, cultural orientation may influence employees in terms of what they perceive as behaviors that are threatening or worthy of investment [13]. For employees who have a high collectivist orientation, they are more willing to utilize personal resources to maintain and facilitate group cohesion, harmony, or cooperation that prioritize organizational goals compared to those with a low collectivist orientation [47]. It is likely that employees high in collectivism would show more positive emotional displays and contribute more energy and dedication to their group and group performance. On the other hand, employees with a low collectivist orientation who focus less on group cooperation [48], will be more likely to utilize affective well-being to achieve personal goals that conserve resources. Consequently, we expect a higher correlation between affective well-being and work engagement for employees with a high collectivist orientation.

**Hypothesis 3** **(H3).** *A collectivist orientation moderates the relationship between affective well-being and work engagement, such that the relationship is stronger for those with a high collectivist orientation.*

Collectivist orientation may similarly moderate the relationship between affective well-being and organizational citizenship behavior. As previously stated, organizational citizenship behavior is conceptualized as voluntary actions due to its definitional emphasis on “extra-role behavior” [49]. Because employees high in collectivist orientation often define themselves in terms of group membership [50] and organizational membership, they would perceive organizational citizenship behaviors as their duty and responsibility, and would view affective well-being as resources willingly used to contribute to displaying organizational citizenship behavior and for the good of the organization. Individuals with a low collectivist orientation might tend to protect one’s own needs and goals by controlling personal costs and gearing resources towards promotion or advancement in the organization [51]. It is possible that employees lower in collectivist orientation will allocate their positive emotions across their own “in-role” domains of work rather than considering the future of the company or the workplace as a whole. This leads us to hypothesize that having a collectivist orientation may positively influence the relationship between affective well-being and organizational citizenship behavior.

**Hypothesis 4** **(H4).** *A collectivist orientation moderates the relationship between affective well-being and organizational citizenship behavior, such that the relationship is stronger for those with a high collectivist orientation.*

## 3. Methodology

### 3.1. Sample and Procedure

We collected data from employees in a large telecom company that provides optical fiber communications for customers (e.g., optical networks, broadband data, fiber optic cables) in central China. This company has 11 departments, such as finance, research and development, manufacturing, and so on. To recruit participants, we contacted the organization’s CEO and managers and informed them about our study.

The study commenced in May 2014, and the participants were required to give informed consent. The data was collected at two points in time to reduce common method bias [52] and improve the methodological rigor in testing the causality of our research model [53]. With the assistance of the human resource management department, 588 employees were invited to participate in a two-wave research effort. Before administering any surveys, employees were informed about the objectives of the study by the HR managers. They were assured that their participation was voluntary and that there was no “undue pressure” to participate. A sealed envelope was sent to 588 employees to complete the first survey which measured affective well-being, their collectivist orientation, and demographic information. After 2 weeks, 461 employees had completed the survey, resulting in a response rate of 78.4%.

We conducted the second wave of data collection by administering the second survey three months later. A sealed envelope was again used to distribute the second survey to the 461 employees who participated in the first wave of the survey. Information was collected on work engagement and organizational citizenship behavior. It is worth noting that the number of potentially available employees was reduced to 311 (i.e., retention rate = 67.5% at Time 2) due to voluntary or involuntary turnover from the company. After removing all incomplete, mismatched, and missing cases, the final sample consisted of 264 employees, making the effective return response rate 84.9%. In this final sample, the participants were 27.48 years old on average (*SD* = 3.96) and had 2.12 years (ranging from 0.5 to 13 years) of organizational tenure. Most participants were male (86%) because the percentage of women in information technology is still small [54]. A majority of the participants graduated from college (76%) or higher than college (20.1%). The analyses were performed using the Statistical Package for Social Sciences version 24.0 (IBM SPSS Statistics 24, SPSS Inc., Chicago, IL, United States) and Mplus 8 (Muthén & Muthén, Los Angeles, CA, USA).

### 3.2. Measures

We followed a translation and back-translation procedure [55] and used a 5-point Likert scale ranging from 1 (*strongly disagree*) to 5 (*strongly agree*). The average mean score was calculated for the four scales.

Affective well-being was assessed with the scale developed by Reker and Wong [56]. The six items refer to thinking about the existence of positive emotions such as happiness, delight, and peacefulness and the absence of negative affect such as fear, anxiety, and depression. A sample item is “I feel that life is worth living”. In the present study, confirmatory factor analysis (CFA) demonstrated that the affective well-being scale had a one-dimensional structure (*χ*^2^ (9) = 22.71, *p* < 0.01; TLI = 0.96; CFI = 0.98; RMSEA = 0.07; SRMR = 0.03), and standardized factor loadings ranged from 0.76 to 0.83. Cronbach’s alpha was 0.90.

Collectivist orientation was measured with two items from Dorfman and Howell [57]. A sample item was “In the work team, group success is more important than individual success”. Because the one-dimensional measurement model with two indicators was a saturated model, it showed a perfect fit, and standardized factor loadings ranged from 0.68 to 0.79. Cronbach’s alpha was 0.70.

Work engagement was measured with the nine-item version of the Utrecht Work Engagement Scale [58]. The work engagement scale consists of three subscales: vigor, dedication, and absorption. Each subscale consists of three items. Sample items were “At my work, I feel bursting with energy” (vigor), “My job inspires me” (dedication), and “I feel happy when I am working intensely” (absorption). In this study, second-order confirmatory factor analysis demonstrated that the work engagement scale had a higher-order latent construct overarching three factors (*χ*^2^ (24) = 65.24, *p* < 0.01; TLI = 0.94; CFI = 0.96; RMSEA = 0.07; SRMR = 0.03). Standardized first-order loadings ranged from 0.77 to 0.88, and standardized second-order loadings raged from 0.91 to 0.97. Cronbach’s alpha was 0.88.

Organizational citizenship behavior was measured with the nine items from Farh et. al. [59]. The scale consists of three subscales: altruism, voice, and conscientiousness. Sample items were “Helps new employees adapt to their work environment” (altruism), “Actively raises suggestions to improve work procedures or processes.” (voice), and “Complies with company rules and procedures even when nobody watches and no evidence can be traced” (conscientiousness). In this study, second-order confirmatory factor analysis demonstrated that the organizational citizenship behavior scale had a higher-order latent construct overarching three factors (*χ*^2^ (24) = 48.28, *p* < 0.01; TLI = 0.95; CFI = 0.97; RMSEA = 0.06; SRMR = 0.04). Standardized first-order loadings ranged from 0.66 to 0.87, and standardized second-order loadings raged from 0.77 to 0.94. Cronbach’s alpha was 0.88.

Gender (0 = male, 1 = female) and organizational tenure (1 = civilian job, 2 = administrative work, 3 = technical work, 4 = market work, 5 = other) are potential predictors of organizational citizenship behavior [60,61]. Gender and job type have also been found to be associated with work engagement [62,63]. Thus, we used gender, job type, and organizational tenure as control variables in our statistical analysis to reduce the possibility of spurious relationships that are based on unmeasured variables.

### 3.3. Preliminary Analyses

In the beginning, we used Mplus 8.0 to perform confirmatory factor analysis (CFA) to verify the discriminate validity of the four constructs in the study. All factors were allowed to correlate with one another in the confirmatory factor analysis. Results revealed that a four-factor model was well-fitted (*χ*^2^ = 695; *df* = 294; RMSEA= 0.07; SRMR = 0.05; TFL = 0.89; CFI = 0.90). We compared the fit of the hypothesized four-factor model with that of a null model, one three-factor models, one two-factor model and one one-factor model. As shown in Table 1, the four-factor model fitted the data better than the other models, providing evidence of the distinctiveness of the constructs of employee perceived affective well-being, work engagement, organizational citizenship behavior, and collectivist orientation.

As recommended by Bernerth, Cole, Taylor, and Walker [64], we analyzed whether it was necessary to control for three socio-demographic variables. By removing control variables uncorrelated with dependent variables, it is possible to avoid potential spurious effects that controls may have when they are significantly related to the predictor, but not the criterion variables [65,66]. The results showed that gender, organizational tenure, and job type did not significantly predict dependent variables, so we did not control for these three socio-demographic variables when testing the hypotheses.

## 4. Results

### 4.1. Descriptive Analysis

The descriptive results are shown in Table 2, with the reliability coefficients shown in brackets. Results showed that affective well-being positively correlated with work engagement (*r* = 0.75, *p* < 0.01), organizational citizenship behavior (*r* = 0.56, *p* < 0.01), and collectivist orientation (*r* = 0.14, *p* < 0.05). Work engagement positively correlated with organizational citizenship behavior (*r* = 0.62, *p* < 0.01) and collectivist orientation (*r* = 0.18, *p* < 0.05). These results are in the expected direction and support the positive relationships between affective well-being, work engagement, and organizational citizenship behavior.

### 4.2. Test of Hypotheses

As the CFA analysis above supported the structural validity of our measurement model, we used observed variables to test the hypotheses [67]. To test our hypotheses, we used Andrew Hayes’ PROCESS (version 2.16.2, 2016) for SPSS to test mediation and moderation (in addition to direct effects). There can be multiple advantages of using PROCESS macro. PROCESS macro offers various complicated regression pathways that structural equation modeling (SEM) programs do not offer. Additionally, it is assumed that not all moderating effects are significant across all ranges of the moderator variable in PROCESS macro [68].

In this study, bootstrapping (5000 iterations) was used to test indirect effects, conditional indirect effects, and to produce 95% bias-corrected confidence intervals. For our mediation hypotheses (Hypotheses 1 and 2) we conducted the analysis using Hayes [68] PROCESS Model 4. For moderation (Hypotheses 3 and 4) we conducted the analysis using Hayes [68] PROCESS Model 1. Unstandardized coefficients are reported.

H1 proposed that affective well-being is positively related to organizational citizenship behavior. We tested our hypotheses using a hierarchical regression analysis run by SPSS. In model 1 of Table 3, the results show that employee affective well-being is positively related to organizational citizenship behavior (*B* = 0.482, *p* < 0.001). Thus, H1 is supported.

H2 argued that work engagement mediated the relationship between affective well-being and organizational citizenship behavior. To test the mediation hypothesis, the PROCESS mediation macro developed by Preacher and Hayes [69] was used. First, we examined the relationship between the independent and mediation variable. The results showed that affective well-being (*B* = 0.752, *p* < 0.001) was a significant direct predictor of work engagement. Second, when affective well-being and work engagement were entered simultaneously, work engagement had a significant effect on organizational citizenship behavior (*B* = 0.438, *p* < 0.001), and affective well-being had a reduced relationship with organizational citizenship behavior (*B* = 0.152, *p* < 0.01). Therefore, work engagement partially mediated affective well-being and organizational citizenship behavior. Furthermore, by using the bootstrapping method for further analyses, we found that affective well-being had a significant direct effect of 0.153 (95% CI = [0.049, 0.256]) and a significant indirect effect of 0.330 (95% CI = [0.227, 0.438]) on organizational citizenship behavior through the mediation of work engagement; not including 0. These results support Hypothesis 2 (see Table 3 and Table 4).

H3 proposed that a collectivist orientation moderated the relationship between affective well-being on work engagement. To support the moderation hypothesis, the coefficients of the interaction terms in the models should be significant. The results showed a significant interaction between affective well-being and collectivist orientation on work engagement (*B* = 0.113, SE = 0.038, *p* < 0.01). H4 hypothesized that collectivist orientation moderated the relationship between affective well-being and organizational citizenship behavior. The results in Table 5 show that the interaction of affective well-being and collective orientation positively predicts organizational citizenship behavior (*B* = 0.084, SE = 0.041, *p* < 0.05).

Further tests of the moderating role of collectivist orientation using the PROCESS bootstrapping approach proposed by Hayes [68] showed that the effects of affective well-being on work engagement and organizational citizenship behavior significantly vary between the high and low levels of collectivist orientation, suggesting that the effects of affective well-being on work engagement and organizational citizenship behavior were smaller at a low level of collectivist orientation than the effect at a high level of collectivist orientation (see Table 6), further supporting H3 and H4. Thus, we conclude that the moderation effects were supported.

**Figure 2:**
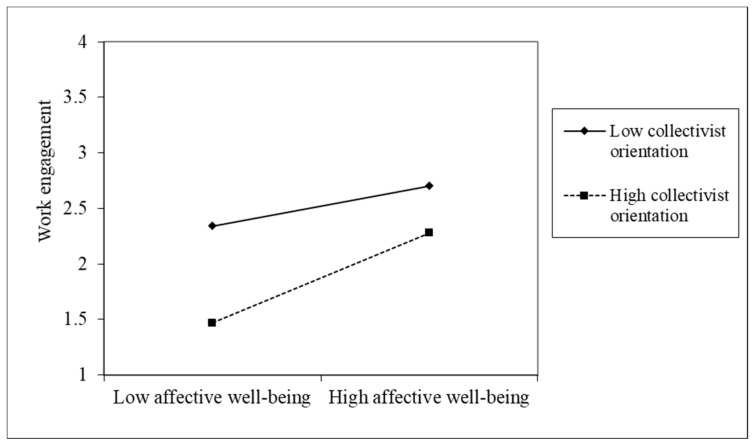
Effect of the interaction between affective well-being and collectivist orientation on work engagement.

**Figure 3:**
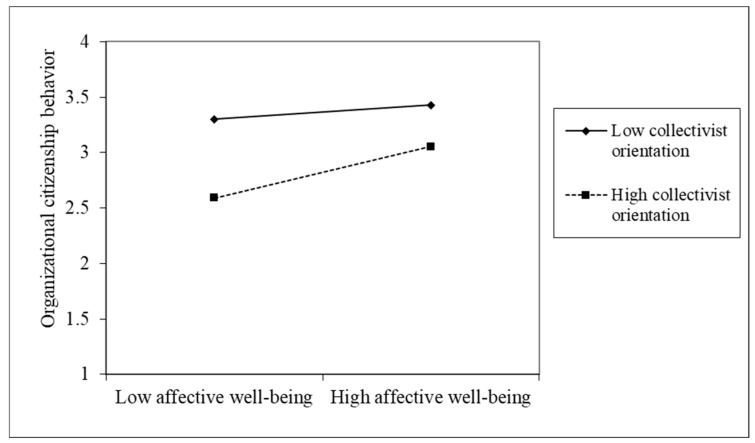
Effect of the interaction between affective well-being and collectivist orientation on organizational citizenship behavior.

To clearly illustrate the moderation effects, the interaction was plotted at one standard deviation below and above the mean of collectivist orientation. As shown in Figure 2 and Figure 3, when collectivist orientation was higher, the relationship between affective well-being and work engagement and organizational citizenship behavior were stronger (the slopes were steeper) than when collectivist orientation was lower.

## 5. Discussion

Using a sample of Chinese employees, this study explored whether and how affective well-being predicts organizational citizenship behavior. This research uncovered three main findings. First, affective well-being plays a significant role in predicting organizational citizenship behavior. Second, work engagement plays a mediating role between affective well-being and organizational citizenship behavior. Finally, a collectivist orientation plays a moderating role in this model by moderating both the relationship between affective well-being and work engagement as well as between affective well-being and organizational citizenship behavior.

Accordingly, our research makes three theoretical contributions. First, our study further establishes the link between affective well-being and performance but more specifically, organizational citizenship behavior. This finding lends support to the basic proposition that “happy” workers are more likely to exhibit extra-role behaviors. Although several researches have investigated relationships between employee well-being (e.g., subjective well-being, happiness) and in-role performance [70,71], organizational citizenship behavior as a dependent variable has been underrepresented (with two exceptions: Gore et. al. [72] and Rego et. al. [73]). We extend previous work by using the Conservation of Resources framework as a theoretical backdrop. We expect that employees’ affective well-being contributes to the conservation and enrichment of resources by employees which then translates to exhibiting more organizational citizenship behavior. Through these findings we expand the knowledge in the emerging field of employee well-being [74,75].

We also explain how affective well-being leads to organizational citizenship behavior via the explanatory mechanism of work engagement. Results of our study show that employee affective well-being has a significant overall main effect on organizational citizenship behavior. Although affective well-being is theoretically relevant to organizational citizenship behavior, it may be more distal and may not exert a strong direct influence on organizational citizenship behavior. We found that affective well-being indirectly contributes to organizational citizenship behavior through promoting work engagement. Consistent with the conservation of resources framework, employees who have positive emotions increase their resources base in the form of work engagement [13,76], enabling them to increase organizational citizenship behavior.

Third, we extend the cross-cultural understanding of affective well-being by examining how having a collectivist orientation shapes the perception of resource loss and gain for employees. By introducing collectivist orientation as a moderator in the relationship between affective well-being and work engagement, and affective well-being and organizational citizenship behavior, this study addresses the limitations mentioned by previous research regarding cultural boundary conditions (e.g., Ayala et. al. [77] and Marescaux et. al. [78]). As a result of the collectivist orientation in Chinese values, Chinese employees value intragroup harmony and affiliation [79]. Consistent with previous studies, our results confirmed the hypothesis that individuals with a collectivist orientation seem to have a heightened sensitivity to affective well-being when it comes to work engagement and organizational citizenship behavior. This suggests that having a collectivist orientation emphasizes the willingness to invest personal resources in terms of work engagement and organizational citizenship behavior. Thus, this study responds to the call of scholars to explore the context and boundary conditions surrounding employee well-being [80,81].

We can also draw some practical implications from our research results. The first implication concerns an investment in employees. As shown in this research, employee well-being is linked to important outcomes such as work engagement and organizational citizenship behavior. As a result, it is important for organizations to provide healthy workplace practices for employees, especially in a country such as China. Enhancing employee control over one’s time at work and supervisor support for workers’ personal life through organizational interventions can contribute to high-tech employees’ well-being [82]. To achieve this goal, organizations should design specific organizational-level initiatives such as flexible work arrangements and mindfulness meditation programs, as well as a focus on work-life balance, workplace safety, job autonomy, and schedule flexibility.

Second, our findings suggest that employees’ work engagement mediates the relationship between affective well-being and organizational citizenship behavior. Thus, besides designing supportive organizational initiatives, organizations should also stimulate employees’ willingness to dedicate physical, cognitive, and emotional resources to their work [83]. To achieve this, managers can help employees to acquire and maintain job resources through job crafting and leisure crafting [84,85,86,87].

Third, our findings concerning the moderating influence of collectivist orientation suggests that organizations should pay particular attention to collectivist values in regards to well-being and its outcomes. It is likely that employees behave differently based on their specific cultural values [88,89] and a collectivist orientation might be important in increasing work engagement and organizational citizenship behavior. Team training and development efforts may benefit from including interventions that help promote collectivist ideals such as team cohesion and cooperation. Promotion of team goals and team norms could also increase collectivist values as well.

The present study has several limitations that should be noted. The first limitation is that although common method bias does not appear to be a problem in our data, the surveys were collected on the same respondents, which may raise some concerns about common method bias [90]. However, we did collect antecedents and outcomes at different points in time so that should have greatly reduced any common method bias. Future research should attempt to replicate our results by using additional sources (e.g., supervisors) to assess work engagement or organizational citizenship behavior. Another limitation is that our two-wave design did not allow us to separate work engagement from organizational citizenship behavior as they were both measured at Time 2. This is important because one could argue that reverse causality might be the case such that organizational citizenship behavior could drive work engagement. Thus, it is not possible to draw causal conclusions regarding the associations between work engagement and organizational citizenship behavior. Future studies should assess variables across three measurement waves to separate affective well-being, work engagement, and organizational citizenship behavior and to be able to draw stronger causal conclusions [91]. Third, our sample was composed of high-tech employees, that is, highly-qualified professional employees. Our findings may not generalize to low-skilled jobs. Moreover, the sample was mostly male. This, combined with the fact that they were highly skilled, may have contributed to their level of organizational citizenship behavior [92]. Thus, our findings need replication using less specialized employees and more gender-balanced samples of employees.

## 6. Conclusions

In sum, the current study adds to the literature on affective well-being, work engagement, collectivist orientation, and organizational citizenship behavior by demonstrating that employee affective well-being is predictive of organizational citizenship behavior and that work engagement is one of the underlying mechanisms through which this relationship occurs. Moreover, having a collectivist orientation is an important moderator, such that when collectivist orientation is high, affective well-being has the highest impact on work engagement and organizational citizenship behavior. Or conversely, when collectivist orientation is low, affective well-being has a lower impact on work engagement and organizational citizenship behavior. 

## Figures and Tables

**Table 1:** Comparison of measurement models.

Models	χ^2^	df	RMSEA	SRMR	TLI	CFI
Four-factor Model	695	294	0.07	0.05	0.89	0.90
Three-factor Model	858	297	0.08	0.06	0.84	0.85
Two-factors Model	1290	299	0.11	0.08	0.75	0.73
One-factor Model	1359	299	0.12	0.08	0.73	0.71

Note: One-factor model, all items were loaded on one factor. Two-factor model, affective well-being, work engagement, and organizational citizenship behavior were loaded on one factor, collectivist orientation. Three-factor model, affective well-being and work engagement were loaded on one factor, collectivist orientation, organizational citizenship behavior. Four-factor model, affective well-being, collectivist orientation, work engagement, organizational citizenship behavior.

**Table 2:** Means, Standard Deviations, and Correlations for Relevant Variables.

Variables		*Mean*	*SD*	1	2	3
1	Affective Well-being	3.53	0.69			
2	Work Engagement	3.56	0.65	0.75 **		
3	Organizational Citizenship Behavior	3.75	0.52	0.56 **	0.62 **	
4	Collectivist Orientation	4.07	0.65	0.14 *	0.18 **	0.10

Note: *n* = 264; ** *p* < 0.01, * *p* < 0.05.

**Table 3:** Mediation effects of work engagement.

Predictors	Work Engagement	Organizational Citizenship Behavior	
	Model 2	Model 1	Model 3
Constant	0.89 ***	2.017 ***	1.627 ***
Affective Well-being	0.752 ***	0.482 ***	0.152 **
Work Engagement			0.438 ***
*R* ^2^	0.625	0.377	0.482
*F*	514.269 ***	186.83 ***	134.548 ***

Note: * *p* < 0.05; ** *p* < 0.01; *** *p* < 0.001.

**Table 4:** Mediation effects of work engagement.

Direct and Indirect Effect of Affective Well-being on Employee Organizational Citizenship Behavior				
	Effect	Boot SE	Boot LLCL	Boot ULCI
Direct Effect	0.153	0.053	0.049	0.256
Indirect Effect	0.330	0.053	0.227	0.438

Note: *N* = 264.

**Table 5:** Moderation effects of collectivist orientation.

Variable	Work Engagement				Organizational Citizenship Behavior			
	B	SE	LLCL	ULCI	B	SE	LLCL	ULCI
Constant	2.198 ***	0.522	1.170	3.226	3.093 ***	0.562	1.987	4.198
Affective Well-being	0.293	0.155	−0.012	0.597	0.148	0.166	−0.179	0.456
Collectivist Orientation	−0.324 *	0.129	−0.579	−0.069	−0.271	0.139	−0.545	0.003
AW × CV	0.113 **	0.038	0.039	0.188	0.084 *	0.041	0.003	0.164
R^2^	0.638				0.385			
∆R^2^	0.011 **				0.008 *			

Note: AW= affective well-being; CV = collectivist orientation; * *p* < 0.05; ** p < 0.01; *** p < 0.001.

**Table 6:** The moderation effects of collectivist orientation.

Collectivist Orientation	Work Engagement				Organizational Citizenship Behavior			
	Effect	Boot SE	Boot LLCL	Boot ULCI	Effect	Boot SE	Boot LLCL	Boot ULCI
Mean −1 SD (3.5)	0.689	0.038	0.614	0.764	0.441	0.041	0.360	0.521
Mean (4)	0.746	0.033	0.680	0.811	0.483	0.036	0.412	0.553
Mean +1 SD (5)	0.859	0.051	0.759	0.958	0.567	0.054	0.459	0.673
